# Capnography and the Bispectral Index—Their Role in Pediatric Sedation: A Brief Review

**DOI:** 10.1155/2010/828347

**Published:** 2010-10-03

**Authors:** Maria Sammartino, Barbara Volpe, Fabio Sbaraglia, Rossella Garra, Alessandro D'Addessi

**Affiliations:** ^1^Department of Anesthesia and Intensive Care, Catholic University of Sacred Heart, 00168 Rome, Italy; ^2^Department of Anesthesia and Intensive Care, General Hospital San Giovanni Calibita, IsolaTiberina, 00186 Rome, Italy; ^3^Urology Department, Catholic University of Sacred Heart, Policlinico A. Gemelli, Largo F. Vito 1, 00168 Rome, Italy

## Abstract

Sedation in children is increasingly emerging as a minimally invasive technique that may be associated with local anaesthesia or diagnostic and therapeutic procedures which do not necessarily require general anaesthesia. Standard monitoring requirements are not sufficient to ensure an effective control of pulmonary ventilation and deep sedation. Capnography in pediatric sedation assesses the effect of different drugs on the occurrence of respiratory failure and records early indicators of respiratory impairment. The Bispectral index (BIS) allows the reduction of dose requirements of anaesthetic drugs, the reduction in the time to extubation and eye opening, and the reduction in the time to discharge. In the field of pediatric sedation, capnography should be recommended to prevent respiratory complications, particularly in spontaneous ventilation. The use of the BIS index, however, needs further investigation due to a lack of evidence, especially in infants. In this paper, we will investigate the role of capnography and the BIS index in improving monitoring standards in pediatric sedation.

## 1. Introduction

Sedation in children is increasingly emerging as a minimally invasive technique that may be associated with local anaesthesia or diagnostic and therapeutic procedures, which do not necessarily require general anaesthesia. During pediatric sedation, the problems to be addressed are linked to a higher risk of upper airway obstruction that can lead to hypoventilation and apnoea. In children who are not always able to cooperate, continuous monitoring of spontaneous ventilation and the “depth” of sedation is therefore essential. In pediatric patients under sedation, standard monitoring requirements, including an electrocardiogram, heart rate, noninvasive blood pressure, pulse oximetry, and respiratory rate, are not sufficient to ensure effective control of pulmonary ventilation and deep sedation. In this review, we will investigate the role of the capnography and the BIS index to improve standards of monitoring in pediatric sedation. Moreover, due to an increasing interindividual variability, maintaining the intended level of sedation is often difficult in children; therefore, the use of cerebral monitoring to indicate the depth of anaesthesia could be particularly useful.

## 2. Capnography

**Figure 1:**
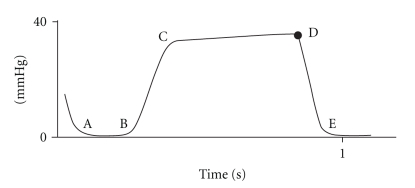
Analysis of the capnograph wave. A: End of inhalation; B: Beginning of exhalation; B–D: Exhalation of alveolar gas; D: End exhalation and point of maximal or highest CO_2_ concentration {end-tidal CO_2_ (EtCO_2_)}; D-E: Inhalation.

**Figure 2:**
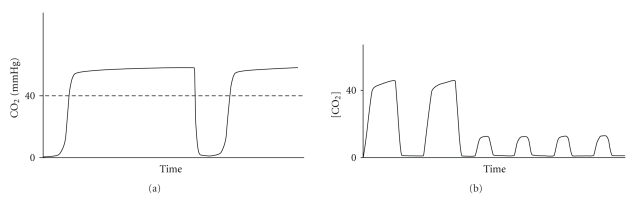
(a) Bradypneic Hypoventilation (type1). (b) Hypopneic Hypoventilation (type2).

**Figure 3:**
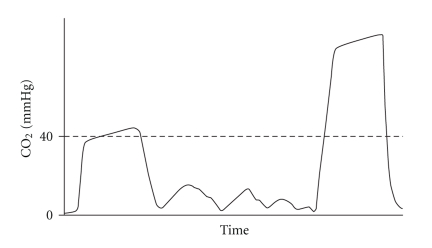
Hypoventilation with a shallow breath followed by a deep breath.

**Figure 4:**
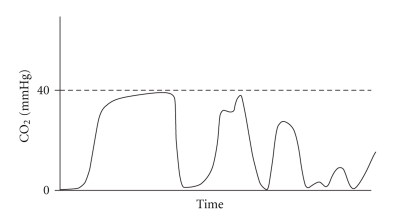
Obstruction of the airways.

**Figure 5:**
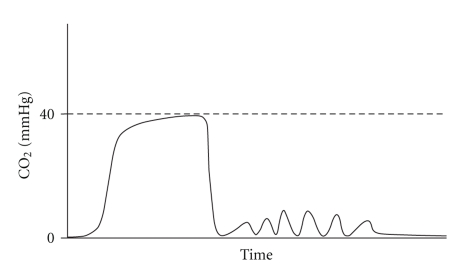
Apnea.

Respiratory monitoring should include the assessment of two components: oxygenation and ventilation. The pulse oximetry has become standard monitoring for assessing oxygenation in conscious patients. End-expiratory carbon dioxide (End-tidal CO_2_) analysis allows assessing of the adequacy of ventilation. The Joint Commission on Accreditation of Healthcare Organizations and The American Academy of Pediatrics [1, 2] have recently recommended capnography monitoring, particularly during sedation. The normal value of End-tidal CO_2_ (EtCO_2_) ranges between 35 and 45 mmHg and indicates the highest concentration of CO_2_ in the expiratory breath measured shortly before the next inspiration. Current standards for respiratory monitoring, including respiratory rate and pulse oximetry, do not always indicate, in real time, the adequacy of alveolar ventilation during spontaneous breathing. In fact, airway obstruction caused by secretions or by the tongue and epiglottis falling back against the posterior wall of the pharynx does not necessarily reduce the respiratory rate. Inspection of the chest, even if performed by an experienced anaesthesiologist, is still a subjective measure and a weak indicator of adequate ventilation. Finally, arterial desaturation due to hypoventilation or obstruction (especially during O_2_ administration) may occur later on. One of the pivotal characteristics of the capnography is its early identification of situations that can cause hypoxia. Therefore, in pediatric patients who have a higher risk of early arterial desaturation due to a reduced Functional Residual Capacity (the volume of air present in the lung at the end of passive expiration), the capnography is a particularly important indicator of altered ventilation [3]. The capnography provides not only the numeric value of EtCO_2_ for each breath, but also a graphical representation of the expiratory phase into its three components (Figure 1). Capnographic Wave A represents the end of the inhalation phase while Wave B represents the beginning of the exhalation phase. Together, A-B indicate Phase I of exhalation and represent an anatomical dead space. Waves B-C represent Phase II: the exhalation of dead space gas mixed with alveolar gas. Wave D indicates the end of exhalation and at the same time the maximum or highest CO_2_ concentrations (EtCO_2_). Phase III corresponds to C-D Waves, that indicates the alveolar gas plateau. Finally, together Waves D-E represent the next inhalation. In Figures 2, 3, 4, and 5, common changes in the trend of the capnographic curve during sedation of a pediatric patient are shown. Recent technological advances have allowed the use of capnography in the management of nonintubated children in spontaneous breathing, even in newborns. In addition, it has also been used in different environments other than in the operating room. Capnography monitors use different types of technology: Mainstream, Sidestream (in use for several years), and the more recently introduced Microstream [4]. In the Mainstream, the sensor is positioned on an airway adapter between the endotracheal tube and the ventilator. In Sidestream monitors, a sample of the gas exhaled by the patient is continuously aspired by a sensor located in the monitor itself. In both types of capnography, humidity and secretions can often occlude the sampling circuit, and the presence of an anesthetic vapor can affect the measurement, especially in pediatric patients because of dilution along the sampling tube. The Microstream system [5] has allowed none of those limitations presented by other systems to be exceeded. In fact, while other capnography systems require a minimum sample of 100–150 ml/min, this system has a sampling chamber of 15 microliters and is reliable even with a capacity of only 50 ml per minute. The Microstream capnography technique can be connected to devices that deliver oxygen through the nose, simultaneously but independently from the sampling of CO_2_, consequently avoiding alterations in measurement. The opportunity to sample both nasal and oral exhaled CO_2_, which is particularly useful in "mouth breather" subjects, is offered by more recent sampling devices. For specific procedures such as a gastroscopy, special devices, that is, the “smart Bitebloc” have been developed. This device provides protection to the gastroscope, and furthermore, facilitates its introduction. In addition, the “smart Bitebloc” simultaneously supplies O_2_ and detects the concentration of exhaled CO_2_. Recent capnography systems are compatible with an MRI (Magnetic Resonance Imaging). 

Recent studies on the use of the capnography in pediatric sedation have assessed the effects of different drugs on the occurrence of respiratory failure. In children sedated in the emergency room for various procedures, opioids combined with midazolam have resulted in a greater increase in EtCO_2_. This is due to probable synergistic effects of opioids/midazolam on respiratory activity compared to the combination of midazolam/ketamine. However, the midazolam/ketamine combination in turn has caused a greater increase in the values of carbon dioxide compared to the administration of midazolam alone [6]. Tobias shows that in pediatric sedation, midazolam 0.05 mg/kg, associated with ketamine 0.5 mg/kg every 1-2 minutes, has a lower incidence of adverse cardiorespiratory events compared to other schemes proposed in the literature, as demonstrated by the low incidence of hypercapnia [7]. There is no evidence of respiratory depression with the use of ketamine 1.5 mg/kg IV. In fact, EtCO_2_ values did not exceed 47 mmHg at any time during the procedure performed on pediatric patients [8]. Despite the evidence-based medicine regarding the use of ketamine, hypercapnia resulting from airway obstruction due to increasing secretions was reported. Yldzdas et al. studied 126 sedated children, divided into 5 groups according to the use of ketamine, midazolam, ketamine/midazolam, midazolam/fentanyl, and propofol. After the administration of propofol, 52% of the patients showed an increase in EtCO_2_ [9]. The authors stressed that hypoxia and hypercapnia were observed in 3.2% of all the patients, whereas hypercapnia alone was found in 16.6%. Therefore, on the basis of the pulse oximetry alone, few patients (4 out of 21) were identified as showing no respiratory depression. Other studies, however, have shown no increase in EtCO_2_ with continuous infusions of propofol [10]. Another area of interest is the comparison of the Capnography with traditional methods such as the pulse oximetry or the monitoring of thoracic impedance to assess phases of hypoventilation during sedation. There are 2 types of hypoventilation that occur during procedural sedation. Bradypneic hypoventilation (type 1), commonly observed with opioids, is characterized by an increased EtCO_2_, an increased PaCO_2_ and a decreased respiratory rate. Bradypneic hypoventilation is graphically represented by a high amplitude and a wide capnogram and can readily be distinguished from hyperventilation which is characterized by increased respiratory rate, low-amplitude, and a narrow capnogram. Hypopneic hypoventilation (type 2) occurs most commonly with sedative hypnotic drugs and is characterized by a normal or decreased EtCO_2_ and an increased PaCO_2_. Hypopneic hypoventilation is graphically represented by a low amplitude capnogram. Both capnography and capnometry have been described the early indicators as much as the only indicators of respiratory impairment [11–13]. Moreover, with Microstream detection systems, the simultaneous administration of oxygen does not reduce the effectiveness of the capnography. 

Koniaris has demonstrated in his analysis of 600 cases of sedations that capnography monitoring can be useful in proving the absence of a drug overdose during endoscopic procedures. In addition, he reiterates the utility of capnography when the patient's respiratory excursions are not visible to the anesthesiologist [14]. This happens for example in an MRI where verbal stimulation, in order to assess the level of sedation, could affect the quality of the examination.

## 3. The Bispectral Index

The Bispectral Index (BIS) is based on an integrated analysis of Electroencephalography (EEG) recordings obtained from electrodes placed in the frontal-parietal areas and expressed numerically on a scale from 0 to 100. The numerical values in sedated patients range from 60 (deep sedation) to 90 (conscious sedation) and are related to the “depth” of anesthesia, but not to analgesia. During pediatric sedation, the BIS index may be particularly important to establish the “depth” of anesthesia, because the patient's anaesthetic plane often becomes unexpectedly deeper, associated with breathing depression. Up to now, monitoring of the levels of anesthesia was evaluated through the use of sedation scales such as the Ramsey and University of Michigan Sedation Scale **(**UMSS), both during the procedure and in the recovery room. Since these scales use verbal or painful stimuli to assess a response, the BIS index appears to be particularly useful in situations such as MRI, SPECT (Single-Photon Emission Computed Tomography), and in the Emergency Room, where the patient cannot be reached by verbal contact or where stimulation can interfere with the outcome of the procedure (e.g., sudden movements caused by stimulation during an MRI that requires absolute immobility). 

As far as the role of the BIS index in children is concerned, the correlation between BIS index and intravenous drugs is valid if propofol, midazolam, thiopental, and fentanyl are used. The correlation is always based on the dose of the drug, the rating scales of sedation and the values of the BIS index [15]. The use of the BIS index in pediatric sedation was proposed when using drugs with long half-life (thiopental, chloral hydrate), either under sedation or in the postoperative period, to avoid dangerous resedation in the recovery room and to achieve a safer discharge [16]. However, this has not been confirmed in recent studies by McDermott and Overly who have shown an insufficient reliability of the use of the BIS index during sedation either with chloral hydrate or with ketamine [17, 18]. Even N_2_O does not show any significant correlation with BIS values [19]. The BIS index is also used to guide the titration of int-ravenous anesthetics such as propofol. During deep sedation, the BIS index can guide the administration of propofol (BIS average 45), avoiding intraoperative awareness or too deep sedation [20]. Sedation with propofol in children is absolutely not a conscious sedation and reiterates the greater utility of the BIS index in guiding the titration of this drug [21]. Other studies have stressed the importance of adjusting the dosage of propofol on the values of the BIS index in order to reduce the incidence of an overdose [22, 23]. The BIS index can be used to guide the administration of anesthesia in children over two years of age. However, in infants under the age of six months, there is no correlation between the values of the BIS index and other measures for assessing the “depth” of anesthesia [24, 25]. The Cochrane review of BIS index monitoring during anaesthesia examined the results of twenty studies. It was found that BIS index-guided anaesthesia reduced the dose requirement of anaesthetic drugs, reduced the time to extubation and eye opening, and reduced the time to discharge. There was a reduction in awareness in high-risk patients. However, all the data regarded the use of the BIS index in the adult population alone [26].

## 4. Conclusions

In the field of pediatric sedation, performed both inside and outside the operating room, the capnography and the BIS index appear very promising in improving safety and providing guidance during the procedures and during the awakening stage. In fact, the capnography should be recommended for the prevention of respiratory complications during sedation in children, particularly in spontaneous ventilation. The use of the BIS index was the only cerebral monitoring validated by the Cochrane company, at least in adult patients. Data for children is copious and seems comforting, but further developments and investigations are necessary due to the lack of evidence, especially in infants.
